# Predicting Visual-Motor Performance in a Reactive Agility Task from Selected Demographic, Training, Anthropometric, and Functional Variables in Adolescents

**DOI:** 10.3390/ijerph17155322

**Published:** 2020-07-24

**Authors:** Marek Popowczak, Jarosław Domaradzki, Andrzej Rokita, Michał Zwierko, Teresa Zwierko

**Affiliations:** 1 Department of Team Sport Games, University School of Physical Education in Wroclaw, I.J. Paderewskiego 35, 51-612 Wroclaw, Poland; andrzej.rokita@awf.wroc.pl; 2 Department of Biostructure, University School of Physical Education in Wroclaw, I.J. Paderewskiego 35, 51-612 Wroclaw, Poland; jaroslaw.domaradzki@awf.wroc.pl; 3 Department of Health Sciences, Wroclaw Medical University, Wybrzeże L. Pasteura 1, 50-367 Wroclaw, Poland; michal.zwierko@gmail.com; 4 Institute of Physical Culture Sciences, Laboratory of Kinesiology in Functional and Structural Human Research Centre, University of Szczecin, al. Piastów 40b, 71-065 Szczecin, Poland; teresa.zwierko@usz.edu.pl

**Keywords:** peripheral perception, team sports, reaction, change of direction

## Abstract

Reactive agility (RA) directly refers to athletes’ visuomotor processing of the specific conditions for team sports. The aim of the study was to identify the factors among age, gender, sport discipline, time participation in a sports activity, reaction time, and visual field which could have an impact on visual-motor performance in RA tasks in young, competitive team sports players. The study included boys (*n* = 149) and girls (*n* = 157) aged 13–15 participating in basketball, volleyball and handball. Anthropometric measurements were carried out, and the Peripheral Perception (PP) test was used to evaluate the visual-motor performance under laboratory conditions. The Five-Time Shuttle Run to Gates test was used to determine the RA. A multiple regression analysis was performed to identify the relationships between the visual-motor performance in an RA task (dependent variable) and the remaining independent variables (continuous and categorical). The findings of the current study indicate that the main predictive factors of visual-motor performance in RA among young athletes are gender (*ß* = −0.46, *p* < 0.000) and age (*ß* = −0.30, *p* < 0.000). Moreover, peripheral perception positively affected the achievements in the RA task in boys (*ß* = −0.25, *p* = 0.020). The sport discipline does not differentiate the visual-motor performance in RA in team sports players in the puberty period.

## 1. Introduction

Visual-motor performance, which is determined in part by central and peripheral vision capacity, has an essential role in open-skills sports such as team sports where players are required to react to stimuli in a dynamically changing and unpredictable environment. Central vision encompasses appropriately directing gazes to an object using the high acuity of foveal vision. In team sports, the ability to attend to other objects (e.g., partners) in the environment using peripheral vision (i.e., without looking directly at them) is equally important. The game situation triggers the processing of information from more than one location using various peripheral visual (e.g., foveal spot, visual pivot, and anchor gaze) or foveal visual (e.g., fixation, quiet eye, saccades, pursuit eye movements) mechanisms that complement each other [1]. Ball sports require the ability to extract and rapidly process dynamic visual features through quickly locating an object, recognizing situations from a dynamic changing environment, and making decisions in a short time [2]. There is evidence that athletes in ball sports exhibit better performance in several visual and motor abilities, including eye-hand coordination, oculomotor function, fusional vergence rate, and reaction time in comparison with a group of individuals without sporting expertise, which suggests an improvement due to the systematic involvement of these skills during sport practice [3,4,5].

Experimental study results have documented that skilled team sports players demonstrate a better capacity to use both their central and peripheral vision when performing the decision-making required in sport-related context tasks [6,7,8]. Moreover, a number of studies have showed that the differences in central and peripheral vision between skilled and less-skilled athletes can also appear in general cognitive tasks without a sport-related context [4,9,10,11], as well as in tests using clinical methodology [12,13]. Studies have demonstrated that visual function can be modified with sport experience as a result of the cognitive and motor demands in a specific sport domain. For instance, Stone et al. [11] investigated basketball players who represent an athlete population that mainly responds to visual stimuli in the upper visual field. They found that the athletes were faster than the non-athletes in response time in both lower and upper visual field tasks, suggesting that the sport experience the athletes had through basketball training significantly shortened their response time, regardless of the visual field. In addition, it has been shown that the faster visuomotor reaction time in athletes in fast-paced sports is mainly determined by visual cortical processes, while motor processes do not contribute to the visuomotor reaction performance [14,15,16]. Moreover, the modulation in visual processing as a result of systematic sports training, which requires rapid detection of and reaction to visual stimuli, has been found in the early stages of sensory processing. Zwierko et al. [17] observed a significant reduction in the visual signal conductivity time recorded at the level of the primary visual cortex over a two-year period of systematic sports training in volleyball players. Considerable changes were also found in visual processing after the stimulation of the peripheral area of the retina. Given the critical importance of dynamic visual input in open-skill sports, one might predict that sport performance might be supported by neuroplastic changes in visual sensory processing modulated by extensive physical training.

A strong link between participation in fast-paced sports, requiring quick responses, and faster visuomotor processes is well documented [7,8,18]. However, there is less information about how these functions change with age and differ across sport discipline, gender, anthropometric variables, and sports experience (years of practice, training per week). The current study was conducted to address this issue by systematically investigating the visuomotor reaction time to visual stimuli, presented at the central and peripheral fields of vision, during a reactive agility task.

Reactive agility (RA), a whole-body movement that requires a change in velocity or direction in response to a non-planed external stimulus, reflects components related to visual sensorimotor processing, as well as representing an essential aspect of motor performance based on an energetic background [19,20,21]. It is generally accepted that agility in sports games is important to optimally prepare players across various stages of training [22,23]. In our opinion, reactive agility tasks directly reflect athletes’ visuomotor processing of important conditions for team sports. Therefore, the current study aimed to investigate the variability of visuomotor performance in a reactive agility task in young talented team sports players. Herein, we attempt to identify the factors among age, gender, sport discipline, time participation in the sport activity, reaction time, and visual field which could have an impact on visuomotor performance. We hypothesized that visuomotor performance in a reactive agility task varies by gender [24] and improves with athletes’ age [25,26,27,28], as physical development is required to master perceptual-cognitive skills and motor demands in the specific sport domain.

## 2. Materials and Methods

### 2.1. Participants

The research was conducted among competitive youth athletes, representing the regional team from Lower Silesia (south-western region of Poland). The participants included 149 boys and 157 girls aged 13 to 15 years. The aim of the regional sports teams is to select young players to the national team in each age category. The athletes represented three different team sports: basketball (boys, *n* = 48; girls, *n* = 45), volleyball (boys, *n* = 47; girls, *n* = 59), and handball (boys, *n* = 54; girls, *n* = 53). The research participants had normal BMIs (girls, 19.88 ± 1.60 kg/m^2^; boys, 19.93 ± 1.52 kg/m^2^). Each participant had a current medical examination to determine their ability to be physically active, as well as determine if they had any refractive errors. All the participants had normal visual acuity and no injuries. The characteristics of the participants are shown in Table 1. All the participants and parents were asked to sign a consent document before testing. All the athletes were thoroughly informed prior to the test about the activity they were to perform and were verbally motivated to complete the task correctly. No training was planned on the day before or the day of testing.

Both parents and coaches had been informed about the purpose of the study and gave permission for the tests. They were also notified that the study was approved by the Senate’s Research Bioethics Commission at the University School of Physical Education in Wroclaw, Poland (17 June 2013), and the procedure complied with the Declaration of Helsinki regarding human experimentation. The study was carried out at the Research Laboratory of the University School of Physical Education in Wroclaw with the ISO 9001: 2009 certification, in cooperation with the Lower Silesian Athletics Federation in Wroclaw, Poland.

### 2.2. Measurements

All the anthropometric measurements were performed by the same experienced researchers. Height measurements for the athletes were conducted with a SECA portable stadiometer 213 (Seca, Hamburg, Germany) with a 1 mm precision. Body weight was measured with a scale, shoeless, and wearing minimal clothes, with the use of stadiometer to the nearest 10 g.

**Figure 1:**
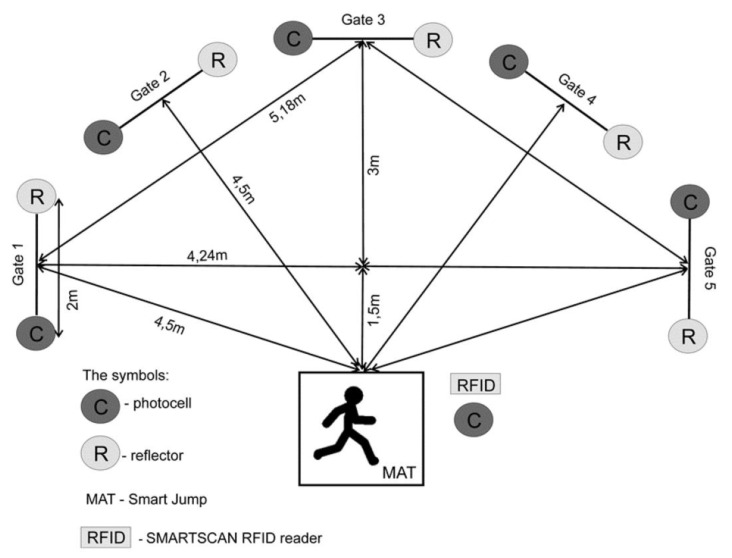
The Five-Time Shuttle Run to Gates test.

The Five-Time Shuttle Run to Gates test allowed the determination of the level of reactive agility. A Fusion Smart Speed System apparatus (Smart Speed, Fusion Sport, Coopers Plains, Brisbane, Australia) was used during the Five-Time Shuttle Run to Gates test. The system compromises gates (each gate is equipped in a photocell with an infrared transmitter and a light reflector), a mat (Smart Jump) integrated with the photocell and an RFID reader for athlete identification, and computer software. The testing apparatus measured running time with an accuracy of 0.001 s. Figure 1 presents the setting of the gates, the mat (Smart Jump), and the RFID reader in this test. The distance covered by the participants in this test was 45 m (Figure 1).

### 2.3. Procedure

The Five-Time Shuttle Run to Gates test [29]: Prior to the test, the subjects underwent a standardized warm-up that lasted 15 min. Afterwards, the researcher discussed the rules of the test and presented its correct execution. Then, each subject received an RFID identification band (containing an individual code), which they put on the wrist of either hand. Before the test, each subject put their identification band on the RFID reader while a green standby light in the head of a photocell connected to the RFID reader was on; this continued until the subject heard a sound signal. Then, all the lights in the gates flashed on and then off again, with only the green standby light remaining lit. The test began with the subject pressing both feet on the center of the mat (Smart Jump). Afterwards, the green light went out, and the light in a randomly selected gate turned on. The subject had to reach the gate, cross the designated line (2 m long) with both feet, and return to the mat (Smart Jump) with both legs, after which another randomly selected gate light lit up. The subject repeated the activity 5 times until after stepping on the mat (Smart Jump) no light signal appeared in the gates. The test data was saved on a handheld device (HP iPAQ 112), where each result was recorded next to the surname of the subject. The test was repeated twice, with a 10 min rest break in between to minimize fatigue. Before proceeding to the test, the subject was familiarized with the procedures by performing a trial (pre-test). The best time of test completion (RA) was used in the analysis of the results.

Peripheral vision and reaction time: The measurement of peripheral perception (vision) was performed using a computer method and a Peripheral Perception (PP) test from the battery of the Vienna Test System (Dr. Schuhfried Medizintechnik GmbH, Moedling, Austria), [30]. The computer-based test consists of two subtasks: a central tracking task and a peripheral perception task. The total field of vision of a healthy person is around 120° in the vertical plane and 200° in the horizontal plane (after the superimposition of the fields of both eyes). The PP test evaluates the ability to receive and process peripheral visual information and maintain central fixation on a target [30]. The test has a very high reliability (r = 0.96).

PP test procedure: The participant (sitting in front of the test apparatus) was tasked with responding in a timely manner to visual stimuli in the form of green glowing vertical diode lines appearing in the lateral field of view on special horizontal LED screens. In the event of the appearance of such a specific stimulus, the participant pressed a foot pedal below the apparatus. At the same time, moving objects—i.e., a ball and a shield—were displayed on the monitor screen, and the subjects were to overlap the objects using panel joysticks. Therefore, the subjects were forced to focus not only on visual stimuli, which were appearing on the right and left sides of their field of view, but also on the moving objects displayed on the monitor in front of them. The device generated a total of 80 pulses (40 on the left and 40 on the right) per time frame. Tracking was controlled by steering a “view-finder” with knobs, such that the view-finder was linked to a red point on the screen. The proper position of the view-finder was confirmed by the flicker of the point. In the test, the system automatically controlled the correct positioning of the head and eyes to the monitor [10,31]. Three parameters were included in the analysis of the results: visual field (FOV_PP_), the median response time of the left and right as peripheral reaction (PR_PP_), and the sum of the correct reactions to the signal (CR_PP_). The FOV_PP_, PR_PP_, and CR_PP_ were determined based on the foot pedal (dominant foot) responses to the green signals (vertical lines).

### 2.4. Statistical Analysis

The Shapiro–Wilk test was used to evaluate the normality of the distribution of the continuous variables. All the variables showed a normal distribution. Descriptive statistics are presented as means, standard deviations, and 95% confidence intervals (CI). The categorical variables were recorded as percentages.

A multiple linear regression analysis was performed to identify the relationships between the visual-motor performance in the reactive agility task (dependent variable) and the remaining independent variables (continuous and categorical). The multiple regression method allows the study of the relationships (influence) of many independent variables with a dependent variable. The advantage is the possibility of using continuous variables, as well as those measured on an ordinal and nominal scale. The significance level was set at α = 0.05. Statistical analyses were performed using the Statistica v.13.0 application (StatSoft Polska, Cracow, Poland).

## 3. Results

Table 1 presents the participants’ characteristics. The young athletes had typical levels of morphological development and normal BMI values for their given age/gender [32]. 

Data from the regression model is presented in Table 2; the model was statistically significant and well suited to the explanatory variables (adjusted *R*^2^ = 0.24, *F* = 10.82, *p* < 0.000). The independent variables included in the model explained 24% of the variability of the visual-motor performance in the RA task. Table 2 also details the results of the testing associations between the individual covariates and the visual-motor performance in the RA. Only three independent variables (gender, age, and training experience) were statistically significant and associated with the dependent variable. The standardized *ß* coefficients suggested that most of the variability of the RA results was explained by gender (*ß* = −0.46, B = −1.24), indicating that boys responded 1.24 s faster than the girls. A slightly weaker effect was observed for age (*ß* = −0.30), wherein increasing the age by 1 year improved the RA performance by 0.5 s (B = −0.48). Although the impact of training experience on the RA results was statistically significant, its strength was negligible (*ß* = −0.11, B = −0.01). Among the morphological features examined, body weight (*ß* = 0.31, B = 0.04) had a strong impact (although not statistically significant) on the RA test result. Specifically, increasing the weight by 1 kg increased the RA test time by 0.04 s. The psychomotor tests performance associations with the RA test were minor.

Due to the substantial influence of gender on the RA performance in the regression model above, further regression analyses were carried out separately for girls and boys. Both the presented regression models (for girls and boys) were statistically significant and well suited to the explanatory variables (adjusted *R*^2^ = 0.14, *F* = 3.43, *p* < 0.000; adjusted *R*^2^ = 0.12, *F* = 2.94, *p* = 0.002, respectively), although the model for the girls is slightly better suited. The regression model for girls explained 14% of the variability of the visual-motor performance in the RA task, while the model for the boys explained 12% of the dependent variable’s variability. Table 3 displays the results of the multiple regression analyses testing associations between the individual covariates and the visual-motor performance in the reactive agility task. The type of team game sport was used as a categorical variable. All the categories were encoded as duplicate variables. The reference group was the volleyball group; thus, we were able to compare the influence of the basketball and handball training on the volleyball training. In the girls’ group, four independent variables were significantly associated with the dependent variable (RA): age, training per week, training experience, and body mass. The standardized *ß* coefficients suggested that most of the variability in the RA performance was explained by the body mass, with a greater body mass being associated with a worse performance (longer time) in the RA test (*ß* = 0.28, B = 0.05). Girls who were more experienced players had substantially better results (shorter time) in the RA test (*ß* = −0.18, B = −0.01). The rest of the statistically significant variables affected the RA results with a similar strength (all *ß* = 0.18, but different B). In the boys’ group, only two independent variables were significantly associated with the dependent variable (RA): age and correct reactions in the PP test. Specifically, the older boys exhibited a better RA performance (*ß* = −0.50, B = −0.79), and the boys who had more correct reactions in the PP test also had better results in the RA test (*ß* = −0.25, B = −0.06). Furthermore, the regression models indicate no effect of sport type on the RA test results among either gender.

## 4. Discussion

The current study examined the impact of the morphological parameters of age, gender, training experience, number of training sessions per week and visual function as assessed by the PP test on the agility test performances in 13–15-year-old team game players. We found that the visual-motor performance in an RA task improves with an athlete’s age. In the current study, a 1-year increase in age resulted in improved agility results of RA by 0.79 s (*p* < 0.000) in boys and 0.26 s (*p* < 0.05) in girls. Our findings confirm previous results in which age significantly influenced the agility test performance in children and adolescents [25,26,27,28]. It appears that the age-associated impacts on RA performance are manifested in individual development and may be attributed to biological maturation [33,34,35,36]. However, studies have shown curvilinear improvements in agility with advancing age—i.e., accelerated improvements for agility in boys are at an age of 9–10 years, whereas in girls this occurs at 9–11 years of age [36]. Furthermore, with age comes technique improvement in reactive agility tests—i.e., mainly the ability to properly accelerate and decelerate, lower the center of gravity in the human body, shorten the step before turning, and respond to an external stimulus [37,38].

Our findings are also consistent with current research on the impact of gender on RA performance. Boys performed better on the Five-Time Shuttle Run to Gates test compared with girls. This is likely a result of differences in the time of peak development in directional change abilities and other motor skills, such as speed and endurance performance. These skills are improved in boys as a result of the onset of puberty [28]. The development of muscle mass, which will affect the strength and speed of movement and change of direction, occurs later. On the other hand, girls go through puberty earlier, reaching their peak development of speed and changes in direction movement. Therefore, female youth athletes may exhibit a stabilization in RA before the final stage of maturation [39].

The better agility performance in boys compared with girls can also be explained by their higher absolute and relative anaerobic power values [40]. Studies examining short-term power output in relation to growth and maturation indicate that the peak and mean power in the Wingate anaerobic test is higher for boys than for girls, and the gender differences increase with age—i.e., the anaerobic power values of boys increased by 121% (peak power) and 113% (mean power), respectively, from age 12 to 17 years, whereas those of girls increased by 66% and 60%, respectively [24].

Our findings revealed no significant impact of height or the type of sport training on the RA performance. However, we did see a significant impact of body mass on the RA performance in girls, with an increase of 1 kg in body mass increasing the task time by 0.05 s. This deterioration in performance is likely related to the lack of power increase with increasing body mass. The participating 13–15-year-old boys were still at the beginning of, or before, the development of muscle mass; therefore, their body mass did not affect the parameters determining visual-motor performance in the same manner [39]. This information may be useful for practitioners designing training for groups of boys and girls at this age. Training for girls should be focused on muscle strength and mass development, which will contribute to the implementation of fast direction changes based on acceleration and braking. Moreover, the management of body composition—e.g., fat-free mass and skeletal muscle mass—may be necessary.

Furthermore, our study results indicate the importance of training experience or the number of training sessions per week on the task performance in girls. Probably, each additional month of training in 13–15-year-old athletes contributes to shortening the test time by 0.01 s (but only in girls). However, the study of the training time volume per week (for example, the hours of training per week) is needed. Similarly, a greater number of exercise sessions per week results in improved visual-motor performance in a reactive agility test in girls. Therefore, additional systematically implemented physical activity for girls in team sports likely contributes to improvements in reactive agility. It should be mentioned that at the time of the study, the young athletes were in a period of motor development stabilization. However, the lack of measurement and analysis of daily physical activity (e.g., undertaken at school or in free time) in our participants may limit the interpretation of this research. Furthermore, information about the type of exercise in sports clubs was not collected. Therefore, we cannot say unequivocally that participation in team sports training impacted reactive agility. Likewise, the type of exercise used in training can affect the test results. However, research conducted by Ryu et al. [8] and Ryu et al. [7] revealed that qualified team sports players (with more training experience) demonstrated a better ability to use visual performance during tasks related to movement than less-skilled players.

In contrast, there was no impact of training experience or the number of exercise sessions per week on the reactive agility performance in boys. This is probably related to the curvilinear course of agility skills observed with age [36]. The boys in the current study were in the initial stages of puberty, wherein muscle hypertrophy (strength and power) is just beginning, and movement coordination and psychomotor processes are still not refined. Therefore, training experience or the number of weekly exercise sessions does not override the high interindividual variability in agility performance in 14–15-year-old boys. Additionally, our results may indicate the importance of the quality and specificity of training classes. The development of RA, as a comprehensive ability consisting of cognitive-perceptual and motor components, requires undertaking physical tasks based on the sprinting ability, speed, and techniques of changing directions and reacting to stimuli from the environment. Without utilizing these types of tasks in training, athletes are unlikely to see any significant changes in RA development and subsequent game performance. A combination of visual skills and plyometric training; motor tasks with a ball similar to the situation in the game—e.g., small-sided games [41,42,43]; and/or agility training is recommended.

In the current study, we found that sport type does not affect the RA performance or associated measures. In studies performed by Šimonek, Horička, and Hianik [22], there was no difference in the horizontal reactive agility between basketballers, volleyballers, and soccer players aged 15–16 years. Therefore, the RA measured using the Five-Time Shuttle Run to Gates test is relevant to various team sports, including volleyball, handball, and basketball. However, these three games are characterized by situations in which the object of observation (ball) is at the height of the torso, head or above the player’s head. In other team sports, such as floorball and soccer, in which the observed ball is usually below the torso, visuomotor performance may differ from that of the athletes in the current study. Additionally, it was noted that subjects who had comparable results in reactive agility also had similar techniques, acceleration, and deceleration. However, it was expected that there would be a visible difference in the reactive agility measures between athletes of various team sports, due to the specificity of movements in the game. Numerous studies have indicated that team sports differ in the number of changes in direction the player performs, acceleration and braking, technique, and the lengths of straight running. Therefore, future research should further examine differences in visuomotor performance between players of different team sports.

The current research looked at the effect of variables characterizing visuomotor performance in the PP test on performance in the Five-Time Shuttle Run to Gates test to assess the RA. The results of the present study showed that the visual field parameters did not affect the RA performance in young athletes. However, it is generally accepted that maturation of peripheral vision occurs until 13 years of age [44]—i.e., simultaneous with the development of agility [36]. Furthermore, Gonçalves et al. [45] found a strong positive correlation between the extent of the visual field and the predicted adult height of soccer players aged 11–15 years. On one hand, the visual field [2] may not be a sensitive discriminator of RA performance. This observation is supported by previous findings where there was no correlation between the extent of the visual field and skill level [10]. On the other hand, it is possible that during RA performance, players used more visual search patterns, based on oculomotor function, to locate the visual stimuli rather than maintaining stable fixation as a base to the peripheral vision. Moreover, the results of perceptual-cognitive studies show distinct differences between experts and novice players in visual search strategies [46,47], suggesting that experts, in contrast to novices, fixate centrally and use peripheral vision to monitor the surrounding areas of the environment. Using alternative tools for oculomotor assessment (e.g., eye-tracker system) may further elucidate the role of visual-motor performance in a reactive agility task.

We also found a positive correlation between the frequency of correct reactions in the PP test and RA performance; however, this relationship was only observed in boys. Boys who had more correct reactions in the PP test performed better in the RA task. Our findings are in line with previous experimental studies that reported gender differences in visual-spatial skills—i.e., it has been observed that boys/men exhibit better spatial perception, spatial visualization, mental turn, and spatial-temporal performance than girls/women, as well as fewer errors when searching for and interpreting stimuli from the environment [48,49,50]. Moreover, during a visual-spatial task, women prefer strategies based on memory function, while men analyze current spatial relationships [51]. Interestingly, it has been reported that sport activity may reduce gender differences in visual-spatial ability. For instance, Notarnicola et al. [48] found no statistically significant gender differences in visual-spatial skills during volleyball and tennis activity, whereas there were significant gender differences in the nonathletic group. Thus, future investigations should monitor the potential benefit of systematic team sports training in reducing gender differences in visual-motor performance.

Our findings may have a contribution in existing theoretical models of agility [52]. According to the constraints-based approach of reactive agility proposed by Jeffreys [53], the positive relationship between correct reactions in the PP test and the RA can be interact to determine the optimal patterns of perceptual constraints. Perceptual and cognitive processes contribute significantly to effective team sports related movement, mainly through the fixation location, recognition of movement patterns, anticipation, and the ability to focus on critical elements contributing to performance. Moreover, it seems to be worthwhile to emphasize that demographic variables, such as age and gender, may constitute the potential constraints that could limit the agility performance.

A primary limitation of this study is that there was no analysis of the biological age of the participants and its subsequent impact on the reactive agility and PP test results. It may be important to establish where the maturation rate is most different between boys and girls and how this relates to visual-motor performance in an RA task.

## 5. Conclusions

The findings of the current study indicate that the main predicting factors of visual-motor performance in reactive agility in young athletes are age and gender. Moreover, peripheral perception positively affects the reactive agility performance in boys. It appears that during puberty, sport discipline does not differentiate the visual-motor performance in RA in team sports players. The practical implications of this study include the potential to modify the training processes for complex motor functions such as agility in relation to the development of future expert athletes. Specifically, the development of RA in team sports during adolescence should be supported by the differentiation of age- and gender-specific training methods. Moreover, the proposal of RA testing is reliable and useful to estimate agility in the puberty period independently of particular sports disciplines in team games. Visual training may be a useful tool for modifying gender differences in visuomotor performance in young athletes.

## Figures and Tables

**Table 1:** Participants’ characteristics by gender and sport categories. Means, standard deviations, and 95% CI are presented.

Variables	Sport	Girls (*n* = 157)			Boys (*n* = 149)		
		Volleyball (*n* = 59)	Basketball (*n* = 45)	Handball (*n* = 53)	Volleyball (*n* = 47)	Basketball (*n* = 48)	Handball (*n* = 54)
Age (yr)	mean ± SD	14.0 ± 0.9	14.2 ± 0.8	14.3 ± 0.8	14.4 ± 0.7	14.4 ± 0.9	14.8 ± 0.8
	(95% CI)	(13.8–14.3)	(14.0–14.4)	(14.1–14.6)	(14.2–14.7)	(14.1–14.6)	(14.5–15.0)
Training per week (*n*)	mean ± SD	3.83 ± 1.18	5.09 ± 1.68	4.06 ± 1.32	5.19 ± 1.71	4.58 ± 0.96	4.41 ± 1.21
	(95% CI)	(3.52–4.14)	(4.59–5.59)	(3.69–4.42)	(4.69–5.69)	(4.30–4.86)	(4.08–4.74)
Training experience (months)	mean ± SD	49.53 ± 19.74	48.96 ± 23.59	47.57 ± 19.83	43.28 ± 18.53	49.27 ± 23.59	44.03 ± 18.79
	(95% CI)	(44.38–54.67)	(41.87–56.04)	(42.10–53.03)	(37.83–48.72)	(42.43–56.11)	(38.91–49.17)
Body mass [kg]	mean ± SD	58.5 ± 6.1	57.0 ± 6.3	55.2 ± 6.9	64.4 ± 9.1	67.0 ± 11.1	63.9 ± 10.2
	(95% CI)	(56.9–60.1)	(55.1–58.9)	(53.3–57.1)	(61.7–67.1)	(63.8–70.2)	(61.1–66.7)
Body height [cm]	mean ± SD	172.6 ± 5.9	169.4 ± 7.3	165.0 ± 6.9	180.2 ± 8.0	182.4 ± 12.2	177.9 ± 10.3
	(95% CI)	(171.1–174.2)	(167.3–171.6)	(163.1–166.8)	(177.8–182.5)	(178.8–186.0)	(175.1–180.7)
CR_PP_ (*n*)	mean ± SD	28.31 ± 4.91	29.27 ± 6.24	28.81 ± 5.56	30.04 ± 5.40	29.06 ± 5.68	28.81 ± 5.85
	(95% CI)	(27.02–29.59)	(27.39–31.14)	(27.28–30.34)	(28.46–31.53)	(27.41–30.71)	(27.22–30.41)
FOV_PP_ (^o^)	mean ± SD	171.46 ± 7.89	172.99 ± 9.46	173.53 ± 6.82	172.31 ± 8.64	171.84 ± 7.05	173.58 ± 8.39
	(95% CI)	(169.40–173.52)	(170.15–175.83)	(171.65–175.42)	(169.77–174.84)	(169.79–173.88)	(171.29–175.87)
PR_PP_ [s]	mean ± SD	0.70 ± 0.07	0.68 ± 0.08	0.70 ± 0.08	0.66 ± 0.08	0.64 ± 0.07	0.65 ± 0.07
	(95% CI)	(0.68–0.72)	(0.65–0.70)	(0.67–0.72)	(0.64–0.69)	(0.61–0.66)	(0.62–0.67)
RA [s]	mean ± SD	19.99 ± 1.15	19.66 ± 1.16	19.58 ± 1.26	18.83 ± 1.36	18.70 ± 1.36	18.55 ± 1.11
	(95% CI)	(19.69–20.29)	(19.31–20.01)	(19.23–19.92)	(18.43–19.22)	(18.30–19.10)	(18.25–18.86)

Note: CR_PP_—correct reaction in the PP test; FOV_PP_—visual field in the PP test; PR_PP_—peripheral reaction in the PP test; RA—reactive agility.

**Table 2:** Individual associations of gender, age, and sport experience covariates; morphological variables; time of reaction; and visual field results with the visual-motor performance in a reactive agility task.

Model	Unstandardized Coefficients	Standardized Coefficients			Sig.
	B	*(ß)*	95% CI		*p*
Gender	−1.24	−0.46	−0.58	−0.34	0.000
Age	−0.48	−0.30	−0.42	−0.18	0.000
Training per week	−0.05	−0.05	−0.15	0.05	0.315
Training experience	−0.01	−0.11	−0.21	−0.01	0.038
Body mass	0.04	0.31	−1.02	1.64	0.645
Body height	0.00	0.01	−1.03	1.04	0.990
CR_PP_	−0.02	−0.07	−0.20	0.06	0.283
FOV_PP_	0.00	0.02	−0.10	0.15	0.719
PR_PP_	0.62	0.04	−0.09	0.16	0.582

Note: CR_PP_—correct reaction in the PP test; FOV_PP_—visual field in the PP test; PR_PP_—peripheral reaction in the PP test; B—unstandardized coefficients; *ß*—standardized coefficients.

**Table 3:** Individual associations of gender, age, and sport experience covariates; morphological variables; time of reaction; and visual field results with the visual-motor performance in a reactive agility test in girls and boys.

Models	Girls					Boys				
	B	*ß*	95% CI		*p*	B	*ß*	95% CI		*p*
Age	−0.26	−0.18	−0.37	0.00	0.049	−0.79	−0.50	−0.73	−0.27	0.000
Training per week	−0.14	−0.18	−0.35	0.00	0.045	−0.03	−0.04	−0.20	0.13	0.674
Training experience	−0.01	−0.18	−0.34	−0.03	0.017	0.00	−0.05	−0.21	0.11	0.548
Body mass	0.05	0.28	0.04	0.51	0.021	0.01	0.11	−0.26	0.49	0.553
Body height	0.00	0.03	−0.22	0.27	0.837	0.04	0.29	−0.08	0.66	0.123
CR_PP_	0.02	0.07	−0.12	0.27	0.463	−0.06	−0.25	−0.46	−0.04	0.020
FOV_PP_	0.00	−0.01	−0.20	0.18	0.929	0.02	0.10	−0.10	0.30	0.308
PR_PP_	2.73	0.17	−0.01	0.36	0.067	−1.47	−0.08	−0.29	0.12	0.423
Reference group: volleyball										
R: Basketball group	−0.01	−0.01	−0.19	0.17	0.911	0.19	0.14	−0.05	0.33	0.149
R: Handball group	0.06	0.05	−0.14	0.24	0.627	0.04	0.03	−0.17	0.22	0.779

Note: CR_PP_—correct reaction in the PP test; FOV_PP_—visual field in the PP test; PR_PP_—peripheral reaction in the PP test; B—unstandardized coefficients; *ß*—standardized coefficients; R—respondent group.
